# Breast Cancer Disparities in Asian Women: The Need for Disaggregated Research

**DOI:** 10.3390/ijerph19169790

**Published:** 2022-08-09

**Authors:** Lauren Fane, Tithi Biswas, Charulata Jindal, Yuk Ming Choi, Jimmy T. Efird

**Affiliations:** 1 MD University Program, Case Western Reserve University School of Medicine, Cleveland, OH 44106, USA; 2 Department of Radiation Oncology, University Hospitals Cleveland Medical Center, Case Western Reserve University, Cleveland, OH 44106, USA; 3 Harvard Medical School, Harvard University, Boston, MA 02115, USA; 4 Signify Health, Dallas, TX 75244, USA; 5 VA Cooperative Studies Program Coordinating Center, Boston, MA 02130, USA; 6 Department of Radiation Oncology, Case Western Reserve University School of Medicine, Cleveland, OH 44106, USA

**Keywords:** Asian women, breast cancer, disaggregation, health disparities

## Abstract

Asian (AZN) women are a heterogeneous group, comprising a wide array of cultural beliefs, languages, and healthcare needs. Yet, studies of breast cancer (BCa) risks and outcomes predominately consider AZNs in aggregate, assuming that the distinct ethnicities have similar disease profiles and homogeneous responses to treatment. This stereotypical portrayal of AZNs as a homogenous group tends to mask disparities. For example, healthcare-seeking behaviors and attitudes of medical providers toward AZN BCa patients frequently differ within this group and from other races. Misconceptions may arise that significantly influence the prevention, detection, treatment, and post-therapeutic care of AZN women. In addition to low BCa screening rates among AZN women, disparities also exist in various stages of BCa treatment—omission of radiation after breast-conserving surgery, less access to hypofractionation, underutilization of hormonal therapy, and higher-cost treatment owing to high HER2+ incidence. In this perspective, we highlight the need for disaggregated research of BCa among AZN women and advocate for comprehensive, culturally sensitive strategies to address health disparities in this priority population. Improving BCa literacy and awareness, access to care, and equitable recruitment into clinical trials are a few amelioratory goals to consider in the future.

## 1. Introduction

Asian (AZN) ethnicities are heterogeneous in many respects—socioeconomic status, education, language competence, cultural values, and genetics. As an illustration of the wide spectrum, Native Hawaiians and Other Pacific Islanders (NHPI) have lower income, less health insurance coverage, and consequently poorer health outcomes than Asian Americans overall [1,2,3,4]. Often, however, research of racial disparities in breast cancer (BCa) combines AZN ethnicities into one group, disregarding distinct characteristics that may aid the diagnosis and management of this malignancy [1,2].

Aggregating AZN groups obscures underlying disparities. For example, the low BCa screening rate in Korean women contrasts the high screening rate of AZNs overall [5]. Additionally, the aggregated 10-year survival of AZN BCa patients is greater than that of NH-White women (91% vs. 83%). Yet, in disaggregated analysis, Native Hawaiian and Other Pacific Islander (NHPI) patients have lower survival than other AZN women, as well as NH-White women [6].

The observed survival differences may be explained by delays in treatment among AZN ethnicities. Specifically, Southeast AZN and NHPI women have longer mean surgery-to-radiotherapy intervals than NH-White women by 6.6 and 10 days, respectively [6]. Similarly, another disaggregated study observed that Chinese women receive surgical treatment sooner than NH-White women, but minority AZN, Hispanic, and Black patients receive surgical treatment later [7]. In contrast, aggregated studies found no delays for surgery or radiotherapy. Some studies even aggregated AZNs with Hispanic and other non-Black racial minorities, exemplifying the need to critically evaluate aggregated studies that find no AZN disparities. Disaggregating AZN groups in research is key to prevent overlooking disparities and opportunities to address them.

Again, “Asian” is an umbrella term encompassing a diverse, heterogeneous, and transnational collection of populations, with varying medical needs and curative propensities [8]. To date, few studies have examined BCa-related health disparities in this population. In this perspective, we aimed to provide a brief synopsis of the literature, emphasizing the cursory nature of the field and the importance of rigorous, disaggregated research.

The predominance of studies discussed and referenced focuses on Asian participants residing in the U.S. “AZN women” in this paper refers typically to Asian Americans and, in some contexts, Asians in general.

## 2. Earlier Age of Diagnosis

BCa presents up to two decades earlier in AZN women. While the peak age of BCa onset for Non-Hispanic White (NH-White) patients is between 60 and 70 years, BCa presents considerably earlier among Asian (AZN) women—between 40 and 50 years [9]. The incidence in AZN countries has increased in recent years and now surpasses rates observed among U.S.-born AZNs—the reverse of what existed prior to the year 2000 [10,11].

## 3. Lower Breast Cancer Screening Rates

AZN women are less likely to receive recommended BCa screening. The longstanding lower BCa incidence in AZN women than NH-White women may be explained by a lower screening rate in the former population [5]. Cancer is the leading cause of death for AZNs, while cardiovascular disease is the leading cause of death for most other racial groups in the U.S. A contributing factor may be that AZNs have lower cancer screening rates [12].

In California, with nearly a third of the AZN population in the U.S., AZN women’s mammography rate (72%) is lower than the state average (79%). AZN women’s BCa screening rate is lower than that of NH-White (81%), African American (83%), and Latina (77%) women. NH-White and African American women have mammography rates higher than the state overall and have already met the national objective in BCa screening rate (81%). However, AZNs have fallen behind [13]. In particular, only half (52%) of Korean women have had a mammogram in the past 2 years [14]. These disparities call for research and interventions to support mammography for AZN women, especially those of Korean ancestry. For example, many Koreans lack health insurance coverage, which likely provides a barrier. Expansions to the Affordable Care Act have provided opportunities to bridge the gaps between recommendations and actual receipt of BCa screening [14].

Despite government and private insurance covering BCa screening for women starting at age 40, younger AZN women (below 40 years) are less likely to receive a mammogram than AZN women aged 50–74. On the other hand, younger NH-White women are more likely to receive a mammogram than older NH-White women. A lower-risk group may be over-screened and receive unnecessary biopsies, while a higher-risk group is under-screened and thus presenting at more advanced stages [15]. Efforts are needed to administer screening according to risk, as well as targeting patient and provider awareness about this topic [16].

## 4. Denser Breasts

Asian women have dense breasts, a BCa risk factor. Having dense breasts is associated with greater BCa incidence, a characteristic independent of reduced BCa detection on dense mammograms [17]. AZN women have greater breast density than NH-White women [18]. When controlling for BMI and age, breast density differences disappeared for all racial groups except AZNs [19]. Yet, AZNs have lower BCa incidence. This seeming paradox highlights that other factors or ethnic heterogeneities not fully adjusted for in these analyses may influence BCa risk [20]. Nonetheless, AZN women overall appear to have greater BCa risk with increased breast density [21,22,23,24]. AZN women and their providers should err on the side of caution and be cognizant of breast density in making decisions about BCa surveillance.

## 5. Sub-Optimal Treatment

AZN women are less likely to receive the recommended therapy at various stages of BCa management. Breast-conserving surgery (BCS) followed by radiotherapy has been the recommended treatment for early-stage BCa since 1990, but many eligible AZN patients still do not receive optimal treatment. AZN BCa patients have the highest mastectomy rates among U.S. women, at the expense of not receiving the recommended therapy (i.e., lumpectomy and radiation) [25]. This difference is especially pronounced in foreign-born AZNs [26].

Factors posited to independently influence choice of surgery (i.e., BCS vs. mastectomy) in AZN BCa patients include age, insurance status, and the time interval between diagnosis and surgery. Older women value cosmetic outcomes less and are more concerned about radiotoxicity. Additionally, a lower level of education, living in rural areas, and lower socioeconomic status are associated with being less receptive to updated research about the recommended therapy providing as much tumor control as mastectomy. Not having adequate insurance coverage influences AZN patients to choose mastectomy because the radiotherapy following BCS is costly. Additionally, a longer wait time to surgery is associated with electing mastectomy. The authors speculated that a longer wait time allowed more opportunity for family and friends to influence patients to choose the traditional, more aggressive surgical treatment (vis-à-vis the perception that it more conclusively prevents recurrence) [27]. Clinicians can make an impact by having conversations with AZN BCa patients about factors guiding their decision-making, assessing their health literacy, and providing patient-focused education.

Some BCa treatment disparities are attributable to AZN patients’ proximity to high-quality facilities. AZN women are more likely than other racial groups to omit radiation after BCS. Living in AZN communities and having low socioeconomic status are associated factors [28]. Other contributing aspects are that AZNs tend to live near hospitals that treat more minority patients, serve many Medicaid recipients, and have low HCAHPS (Hospital Consumer Assessment of Health Care Providers and Systems) quality scores [29]. Disparities also exist in hypofractionation, a strategy that compresses radiotherapy treatment into higher doses and fewer appointments. This regimen reduces the patient burden of frequently commuting to radiotherapy sessions. AZN women are less likely to receive hypofractionation than NH-White women, which is attributable to them living farther from facilities that use hypofractionation [30].

AZN patients also are less likely to receive breast reconstruction. Patients for whom English is not their primary language have lower odds of being informed of reconstruction by a doctor. This disparity calls for increased efforts in interpretation services to inform AZN BCa patients of the option of reconstruction, immediate reconstruction, and if their insurance adequately covers the procedure [31].

## 6. HER2-Positive Breast Cancer

Multiple AZN ethnicities have an increased risk for HER2-positive (HER2+) BCa [32]. This biological subtype is aggressive and has poorer survival than more common hormone-receptor-positive types of BCa [33]. Additionally, HER2+ therapy is more expensive than those of other BCa subtypes, owing to the length of treatment and cost of the cancer drug (e.g., trastuzumab) [34]. High treatment costs place a financial burden on states’ public healthcare systems, which provide care for many AZNs [33].

Of patients diagnosed with hormone-receptor-positive BCa in the Kaiser Permanente North California (KPNC) system, Chinese American and Hispanic women were less likely to initiate adjuvant hormonal therapy than NH-White women. Since all of these participants were insured, access to care is not likely to account for the differences, and other reasons should be explored (e.g., low utilization rates, poor health literacy, language barriers, and the use of non-Western therapies). Interventions aimed at those with highest risk of not using hormonal therapy are warranted, especially enhanced education about the treatment benefit [35].

## 7. Racism and Discrimination

AZN patients experience discrimination from healthcare providers, which influences their healthcare seeking/avoidance behavior and quality of care. They tend to self-blame and exhibit belittling coping behavior when experiencing discrimination. This coping behavior is based on believing that minority groups must work harder to achieve success or overcome everyday challenges. For instance, with the goal of receiving better care, AZN BCa patients reported often presenting positive images of themselves to avoid stereotypes. In response to poor care or communication difficulties with providers, AZN BCa patients often faulted their own inadequacies, such as low language proficiency or education. Furthermore, language barriers were most reported by AZN BCa patients, as few providers speak AZN languages and interpretation services are limited [36]. AZN women, regardless of education level, reported lower perceived quality of care than NH-White women [37].

A survey of Korean American women in Chicago suggested that low mammography rates can be attributed to perceived discrimination in health care, as well as distrust in providers and the health care system [38]. Trust can be improved through educational interventions. Translation services for AZN patients should be enhanced, especially in-person versus telephone/virtual interpreters. Employers and medical education should encourage clinicians to strengthen/acquire medical AZN language skills.

## 8. Immune Checkpoint Inhibitors

Immune checkpoint inhibitors (ICIs) are an important new class of therapies for cancer. While BCa has traditionally been considered not immunogenic, recent molecular studies have found otherwise, and ICI clinical trials for BCa have begun [39]. The HER2+ subtype, which has a higher incidence in AZN women, has durable anti-tumor activity, specifically those expressing the ICI target PD-L1 [40]. In general, AZN breast cancers were found to have more infiltration of immune cells into tumor tissues [41]. These findings suggest that Asian BCa patients may be more likely to respond to ICI therapy. Indeed, a meta-analysis of ICI clinical trials (mostly non-small-cell lung cancer and gastric cancer) reported that AZN patients have a significantly improved survival benefit than non-AZN patients receiving PD-1/PD-L1 inhibitor-based therapy [42].

On the other hand, a study of melanoma found that AZN patients have lower expression of some ICI therapeutic targets: PD-L1, CTLA-4, and IDO-1 [43]. Conceivably, BCa in AZN women may have similarly reduced levels, which should be explored in future research.

## 9. Conclusions

In summary, several points are worth emphasizing. Future research for new BCa treatments should proportionately represent AZN patients, disaggregate AZN ethnicities, and increase attention on potential disparities. Given that they present with BCa at younger ages and manifest low screening rates, AZN women should be informed of their greater need for earlier mammograms and be encouraged to adhere to regular screening. Since AZN women tend to have dense breasts, providers should emphasize this risk factor for BCa and perhaps recommend more advanced imaging modalities (e.g., MRI and tomosynthesis for early lesions that may not be detected with traditional mammography) [44,45,46].

Disparities exist in various stages of BCa care, including the omission of radiation after breast-conserving surgery, less access to hypofractionation, underutilization of hormonal therapy, and more expensive treatment owing to higher HER2+ incidence. Additionally, AZN women are most likely to choose mastectomy over recommended therapy, which highlights the need for targeted patient education. Likewise, language barriers remain a concern and emphasis should be placed on promoting conversation about treatment options, including reconstruction. The research and clinical gaps realized from past experience can be leveraged to improve BCa care and surveillance for this fast-growing but diverse priority population.

## Data Availability

Data sharing not applicable.
